# Supplementary material for: Are carbon emissions trading and green financial instruments synergistic? -Comprehensive quantitative research based on content analysis
Source: PLoS One. 2024 Mar 7;19(3):e0298601. doi: 10.1371/journal.pone.0298601 (PMC10919601; doi:10.1371/journal.pone.0298601)
Supplement: S1 Data — (ZIP) [file pone.0298601.s001.zip › Data/地级市Z_F工作报告.zip!/╕ú╜¿/╕ú╓▌╩╨/└┤╫╘╣1⁄2╓┌║┼í╛┬φ┐╦╩2╛▌═°í┐_2013.docx]

各位代表：

　　现在，我代表福州市人民政府向大会作政府工作报告，请各位代表审议，并请市政协各位委员和其他列席人员提出意见。

　　一、2012年工作回顾

　　2012年，在省委、省政府和市委的正确领导下，我市各级政府认真贯彻落实党的十七届五中、六中全会和十八大精神，坚持稳中求进，着力稳增长、扩内需、转方式、调结构、扶企业、惠民生，推动经济社会平稳较快发展。除外贸出口由于外需严重不足负增长14.1%外，总体上较好完成了市十四届人大一次会议确定的各项任务。预计全市生产总值4203亿元，增长12%；公共财政总收入597.39亿元，增长18.1%，其中，地方公共财政收入382.01亿元，增长19.4%；全社会固定资产投资3264亿元，增长20%；按验资口径实际利用外资13.3亿美元，增长4.2%；社会消费品零售总额2246亿元，增长18.4%；居民消费价格总水平上涨2.2%；城镇居民人均可支配收入29400元，增长13%；农民人均纯收入11700元，增长16%；城镇登记失业率2.4%；人口自然增长率6.97‰；省下达的节能减排任务预计可以完成。一年来的主要工作及成效是：

　　(一)产业转型升级步伐加快。现代农业加快发展，粮食产量保持稳定，农林牧渔业总产值630亿元，比增4.9%。工业新增长点加快形成，规模以上工业总产值5870亿元，增长15.5%，建成投产99项重点工业项目。海西高新技术产业园整合、建设步伐加快，闽台(福州)蓝色经济产业园、临空经济区等新兴产业园区建设前期工作扎实推进。商贸、物流、金融等服务业快速发展，福州成为全国商务诚信建设试点城市，海峡电子商务产业基地入选首批国家电子商务示范基地，渣打银行等世界知名金融机构落户福州。一批文化旅游综合体项目加快实施，福州入选首批国家智慧旅游试点城市。闽台(福州)文化产业园被评为国家级文化产业试验园区。成功举办第87届全国糖酒商品交易会等76场全国性、区域性展会。

　　(二)经济发展后劲显著增强。成功举办一系列投资推介、对接洽谈活动，签约“三维”项目364项、总投资约3111亿元。“五大战役”完成投资1537.7亿元，为年度计划的129.4%；实施市级重点项目622项，完成投资1566.04亿元，为年度计划的125.8%。强化创新驱动，新增15家省级创新型企业，新设立7家省级企业技术中心、11家省级企业工程技术研究中心和4家行业技术创新中心，新认定34家院士(专家)工作站，福州获评首批国家知识产权示范城市，并获批建立全国首个综合性的国家引智试验区。组织开展首届政府质量奖评选活动，新增8件中国驰名商标和2件国家地理标志证明商标。

　　(三)大开放新格局不断拓展。福州被评为中国钟表产业外贸转型升级专业型示范基地，江阴港区获批成为国家汽车整车进口口岸。大力提高招商实效，积极实施“回归工程”，新批千万美元以上外(台)资项目40项。先后与5家中直、省直部门及36家央属、省属企业，6所高等院校，13家金融机构签订战略合作协议。成功举办央视中秋晚会、福州论坛—创业与创投、中国(福州)智慧城市高峰论坛等一批国际性活动，有效提升福州的全球知名度与影响力。切实加强榕台交流合作，台商投资区扩区获得国务院批准，全国首个涉台商品原产地办公室在福州设立，赴台个人游顺利实施，成功举办第十四届海交会以及海峡两岸合唱节、渔业周暨渔博会等对台特色交流活动。积极支持平潭开放开发，闽江调水、电力专线上岛、长平高速公路等重大项目启动建设。榕港榕澳合作进一步深化，福州成为全国落实CEPA示范城市。新一轮援疆、援藏、援宁等工作扎实推进，侨务、外事、异地商会等工作取得新的成果。

　　(四)重点领域改革持续深化。国有资产运营监管水平进一步提高，“大国资、全覆盖”监管格局初步形成。BT、BOT等投融资建设模式得到广泛应用，道路清扫保洁等领域市场化运作启动试点。进一步简政放权，取消、调整、合并216项行政审批事项，下放117项行政事权。县(市)区政府机构改革全面完成，事业单位分类改革稳步推进。“营改增”、“公务卡”等改革试点顺利开展。市属文化单位改制、公立医院改革、中考中招改革等工作扎实推进，福州获评全国文化体制改革工作先进地区。“135”社区党建工作模式被列为全国社区建设典型。农村综合改革不断向纵深拓展，县(市)区对口协作全面展开。

　　(五)城乡环境面貌明显改观。加快提升城市建设发展水平，福州大都市区发展规划、马尾新城总体规划基本编制完成。马尾新城建设全面启动，螺洲大桥等项目基本建成。鼓岭综合整治及保护开发成效显著，获评省级旅游度假区。打响城乡环境综合整治大会战，造林绿化38.9万亩，新增300万平方米城市公共绿地，中心城区75条内河基本完成清淤，西湖和晋安河、新西河、东西河、新店溪等内河综合整治初见成效，光明港至森林公园绿道等一批项目建设完成。着力缓解群众出行难问题，地铁1号线全线动工，新建和改扩建市政道路22条，实施74公里市政道路亮灯工程建设，新增公交车辆469辆，新辟和优化公交线路79条。道路交通安全、违法建设、户外广告、违法采砂等专项整治活动深入开展，“数字城管”系统投入试运行。福莆宁同城化发展框架协议签订实施，“旅游一票通”等工作取得较好成效。实施重点节能减排项目306项，开展大气PM2.5监测，成功创建5个省级以上生态县(市)区、59个省级以上生态乡镇，城市环境综合整治定量考核成绩持续位居全省前列。

　　(六)新农村建设扎实推进。坚持以工促农、以城带乡，积极促进强农惠农富农。福州成为全国“农超对接”试点城市，并获得“中国鱼丸之都”、“中国纯天然远洋捕捞产品产销基地”等称号，福州茉莉花茶获评“世界名茶”，福州茉莉花种植与茶文化系统被列为中国重要农业文化遗产候选项目。启动建设24项重大水利项目，治理水土流失13.8万亩。13个省、市试点小城镇建设取得较好成效。新农村建设“百村竞赛”等活动深入开展，培育出晋安宜夏、福清溪头、长乐青山、永泰芋坑等精品示范村。在全省率先实现行政村体育设施全覆盖，新建改造农村公路280公里，新增更新农村客车108辆，新建农村户用沼气池2000口，新建改建农村卫生户厕5888户，改造提升乡镇卫生院21家和村卫生所1024个，完成1058个村广播电视村村通建设，解决50万农村人口饮水安全问题，实施“造福工程”搬迁3100人。

　　(七)各项社会事业长足进步。不断巩固提升文明城市创建成果，城市文明程度指数、未成年人思想道德建设工作测评成绩分别位居全国省会、副省级文明城市第5位和第4位，创历史最好名次。加快发展各级各类教育，新建和改扩建中小学50所、公办幼儿园50所，完成400所义务教育标准化学校创建任务，扩容10550个中小学学位，西湖国际学校建成投入使用，在全国率先开办服务自闭症儿童的福州星语学校。进一步提升市级专科医院专业化水平，新增住院床位1600张，建成市级卫生信息综合平台。三坊七巷、“海上丝绸之路：福州史迹”入选中国世界文化遗产预备名录，福州获得“中国寿山石文化之都”、“中国脱胎漆艺之都”等称号。国家级文艺创作基地和全国首家篆刻印吧“左海印吧”落户三坊七巷，《百年上下杭》获得中国电视纪录片十优作品奖，新福州人歌手大赛获评全国农民工文化服务示范项目。成功举办第23届市运会和环福州·永泰国际公路自行车赛等活动，在伦敦奥运会上我市运动员取得优异成绩。低生育水平保持稳定，人口计生服务质量进一步提高。民族宗教、民政、新闻出版、广播影视、地方志、档案、妇女儿童、老龄、残疾人等事业加快发展。国防动员、民兵预备役建设、海防、人防等工作扎实推进，福州获得全国双拥模范城“七连冠”。

　　(八)人民生活水平稳步提升。坚持以人为本、改善民生，集中财力多办惠民实事。全市各级财政用于民生支出292.08亿元，占公共财政支出的71.3%。年初确定的20件93项为民办实事项目全面完成。城镇新增就业14.56万人，转移农业富余劳动力5.37万人，超额完成年度目标任务。城乡居民养老保险制度实现全覆盖，新农合大病保障机制基本建立，城乡低保、企业离退休人员养老金、城乡居民基本医疗保险补助标准进一步提高。市场供应基本做到量足价稳质优，主要食品安全检测指标全部达标。居家养老服务实现社区全覆盖。实施危旧房(棚屋区)改造180万平方米，综合整治老旧住宅小区75个，基本建成保障性住房3.43万套，超额完成省下达的任务。“平安福州”建设成效明显，人民群众对社会治安满意率达95.05%，安全生产事故死亡人数下降25.9%。有效解决了一大批信访积案和历史遗留“两权证”办理等热点难点问题，人民调解、行政复议、法律援助、社区矫正等工作不断加强，省会中心城市保持和谐稳定。

　　(九)政府自身建设切实加强。大力弘扬“马上就办”的传统，不断改进作风、提高效能、优化服务。市行政服务中心当场办结率72%，群众满意率99.99%，在全省率先推行了市县联动服务。“三服务”、“让人民满意”媒体直播民主评议政风行风等活动取得良好成效。政府信息公开和“中国福州”门户网站绩效考核位居全国前列，开通“福州发布”政务微博群。在全省率先开展廉政风险防控工作，行政监察、审计监督不断加强，惩治和预防腐败体系进一步完善。提请市人大常委会审议地方性法规草案5件，制定政府规章和规范性文件25件。认真执行人大及其常委会决定决议，自觉接受人大及其常委会的法律监督、工作监督和政协的民主监督，办复611件省、市人大代表建议、批评、意见和561件省、市政协提案，满意率分别为96.4%和99.8%。

　　各位代表，回顾过去一年的工作，我们取得的成绩来之不易。这是省委、省政府和市委正确领导的结果，是全市人民团结拼搏、攻坚克难和各方面大力支持的结果。在此，我代表市人民政府，向全市人民，向人大代表、政协委员、各民主党派、工商联、各人民团体、离退休老同志和社会各界人士，向中直、省直机关企事业单位和驻榕部队、武警官兵、公安政法干警，向关心支持福州发展的港澳台同胞、海外侨胞和国际友人，表示衷心的感谢！

　　在看到成绩的同时，我们也清醒地认识到存在的问题与不足，主要是：思想观念、体制机制某些方面还不适应大开放、大发展的要求；经济总量不够大，综合实力不够强，产业结构不够优；城乡规划建设管理存在薄弱环节；城乡发展、区域发展不平衡；民生保障水平还有待进一步提高；城乡生态环境保护任务较为艰巨；维护社会稳定压力加大；机关作风和效能建设仍需加强，一些干部的思想作风、能力素质与新形势新任务不相适应。我们要正视这些问题，采取有力措施，认真加以解决。　二、2013年工作安排

　　各位代表，2013年是全面贯彻落实党的十八大精神的开局之年，也是我们在圆满实现“3820”工程宏伟目标的基础上，认真实施“十二五”规划、力争率先全面建成小康社会的重要一年。我们要大力传承弘扬“3820”工程战略精髓，牢牢把握稳中求进的总基调，以提高经济增长质量和效益为中心，进一步开拓创新、全力以赴做好各项工作，加快推进闽江口发展区建设，实现经济持续健康发展和社会和谐稳定。

　　今年全市经济社会发展主要预期目标是：地区生产总值增长11.5%；地方公共财政收入增长13%；全社会固定资产投资增长12%；外贸出口增长5%；按验资口径实际利用外资增长5%；社会消费品零售总额增长15%；居民消费价格总水平涨幅控制在3.5%左右；城镇居民人均可支配收入增长12%，农民人均纯收入增长13%；城镇登记失业率控制在3.5%以内；人口自然增长率控制在7.6‰以内；单位地区生产总值能耗下降3.43%，确保完成减排“十二五”中期目标任务。

　　为实现上述预期目标，重点抓好以下八个方面工作：

　　(一)以解放思想为先导，坚定不移地深化改革开放。

　　更加积极地推进重点领域改革。完善国有资产管理体制，做大做强市属投融资平台，培育若干家上规模的国有企业集团。大力支持与引导非公有制经济发展，进一步拓宽民间资本投资渠道。推行政府购买公共服务等模式，在五城区全面实施道路清扫保洁、内河保洁市场化运作。继续鼓励和引导企业上市。完善公共财政体系，抓好“营改增”试点工作，全面实施“公务卡”制度改革。认真做好简政放权工作，推进事业单位分类改革，加强市、县行政服务中心建设，建立“一站式”为全市人民群众集中便捷办理各种民生事项的市民服务中心。深化教育、文化、卫生等社会事业领域各项改革，规范发展民办教育、文化、医疗机构。创新农业生产经营体制机制，推进农村土地承包经营权流转平台建设。加快改革户籍制度，有序推进农业转移人口市民化。

　　更加积极地实施大开放战略。继续加强“三维”对接，深入实施“回归工程”，大力提高对接项目的履约率和开工率。提升海交会办会水平与实效，积极争取举办一批国际性会议、活动。加快发展现有各类国家级开发区、投资区，推进产业链招商、以商招商。争取罗源湾港区尽快获批对外开放。进一步落实侨务政策，创新侨务机制，用好侨务资源，发挥侨智侨力。加强榕港榕澳在金融、物流、旅游等方面的合作。不断拓展国际友城合作及泛珠三角、闽浙赣皖、闽东北等区域协作、山海协作，继续做好对口支援新疆、西藏、宁夏等工作。

　　更加积极地推动榕台交流合作先行先试。认真落实《海峡两岸投资保护协议》，支持在榕台资企业发展壮大和转型升级，加快推进台商投资区、闽台(福州)文化产业园、海峡两岸(福州)农业合作试验区建设发展，加快建设两岸金融中心。大力发展海峡旅游，争取开辟黄岐至马祖海上航线。支持建设海峡邮政枢纽中心、邮政电讯直通口岸。深化榕台全方位、常态化交流合作，办好海峡版权(创意)产业精品博览会等对台特色交流活动。

　　更加积极地支持和对接平潭开放开发。

　　大力支持平潭建设两岸同胞共同家园，扎实推进闽江调水、入岛通道等项目建设，继续在科技、教育、卫生、人才培养、公共服务等方面为平潭开放开发提供良好服务。积极对接平潭至台中等海上直航航线，着力打造两岸人流、物流的“黄金通道”。主动争取平潭综合实验区优惠政策的辐射，促进平潭与周边地区有机联动发展。

　　(二)着力扩内需稳外需，促进经济持续健康发展。

　　积极扩大城乡居民消费。拓展教育、文化、旅游、健身、休闲、养老、家政等消费，鼓励发展电子商务、信贷消费。促进房地产市场平稳健康发展，推动装修、家电、家具等大宗商品消费。积极申办全国性、区域性展会，大力发展会展经济。完善便民消费体系，推进商务诚信建设、“农超对接”等工作，坚决治理“餐桌污染”，严厉打击侵害消费者权益的违法行为。

　　大力实施投资拉动。加大现代产业体系、海洋经济、环境整治、农田水利、公共服务、基础设施建设等领域投资力度，进一步打好“五大战役”，加强重点项目建设，增加并引导好民间投资。初步安排“五大战役”项目781项，总投资8821亿元，年度计划投资1337亿元；市级重点项目300项，总投资9688亿元，年度计划投资876亿元。

　　努力稳定和拓展外需。加强电子信息产品、船舶、家具及装饰品、钟表等重点出口商品基地建设，推进加工贸易转型升级，提高服务贸易出口比重。充分发挥福州保税港区、江阴国家汽车整车进口口岸政策功能优势，积极发展国际中转、采购、配送和转口贸易。扩大重要装备、先进技术和紧缺资源进口。积极支持有条件的企业“走出去”，进一步争取订单、开拓市场。切实帮扶出口企业更好地防范经贸风险、应对贸易壁垒。

　　(三)加快调整优化产业结构，大力发展壮大实体经济。

　　加快制造业转型升级。动工建设恒申合纤三期、中航国际飞机制造等53个项目，推进中石油钢管制造、兆元光电等103个在建项目建设，争取建成天辰耀隆己内酰胺、科立视触控屏等70个项目，力争新增1个千亿产业集群和3家百亿企业。发展提升金山、福兴、江阴、滨海、青口等工业园区，加快建设可门临港工业集中区、闽侯经济开发区、闽清白金工业区及罗源湾、长乐宝钢不锈钢深加工产业园等一批新兴特色产业园区。实施163项工业转型创新重点项目，加强传统产业技术改造。大力实施品牌战略，培育、扶持和保护名优地产品。加大对中小企业扶持力度，切实帮助企业解决融资、用工、用地等实际问题。

　　加快高新技术产业培育壮大。着力发展新一代信息技术、高端装备制造、生物与新医药、新材料、新能源、节能环保、物联网等战略性新兴产业，认真实施新能源汽车、LED等市场应用示范工程，力争新增50家以上高新技术企业，高新技术产业产值突破2500亿元。加快福州高新区发展，进一步提速建设海西高新技术产业园，积极推进海峡软件新城、仓山科技园区、中海创(永泰)智慧科技园区建设。创建物联网国家级技术研发中心，力争福州经济技术开发区成为国家级物联网产业园。

　　加快服务业提质增效。加速推进海峡金融商务区、闽江北岸中央商务区、南台岛三江口商务区建设，建成苏宁广场、泰禾城市广场等一批城市综合体，进一步做大做强总部经济、楼宇经济。加大台江海峡电子商务产业基地二期建设力度，做好国家电子商务示范城市建设和鼓楼区国家服务业综合改革试点工作。加快建设海峡钢贸城、海峡商品交易中心，提升海峡汽车文化广场及水产品、农副产品等批发交易中心。积极推进物流服务平台建设，大力发展第三方物流、冷链物流和城市配送体系。支持福建海峡银行、福州农商银行引进战略投资者，争取更多的世界知名金融机构落户福州，鼓励发展创业投资公司、小额贷款公司、村镇银行等金融机构，加快打造海西现代金融中心。着力争创中国服务外包示范城市。全面启动临空经济区和机场保税物流园区建设，争取福州航空有限责任公司尽快获批运营。

　　加快旅游、文化创意产业做强做优。打响闽都文化、温泉养生、休闲度假、生态旅游等旅游品牌，推进三江口、东雁、桂湖、东壁岛、鼓岭、云顶、下沙等一批在建拟建的文化旅游综合体和高端旅游项目建设，力争三坊七巷成功创建国家5A级旅游景区。打造“船游福州”特色旅游产品，加快闽江北港及连江、罗源等地游艇项目建设。推进国家智慧旅游城市试点工作，力争实现中心城区三环以内旅游标识全覆盖。培育壮大七大文化创意产业，推进船政文化城、国家广告产业园、海峡文化创意产业基地等项目建设，提升福州国家动漫产业实验园发展水平。

　　加快“海上福州”建设发展。坚持“三群”联动，大力推进江阴海港新城以及罗源湾、可门等港口新城建设。支持发展远洋渔业和海产品精深加工，推动马尾、福清、长乐、连江等地水产品加工基地建设。积极发展临港工业、港口物流、海洋运输、滨海旅游、海洋生物医药等“蓝色产业”，加快建设闽台(福州)蓝色经济产业园、粗芦岛海洋工程装备制造业基地。加强福州港泊位建设、航道疏浚、航线开辟，积极稳妥推进罗源湾养殖清理。

　　(四)认真实施创新驱动发展战略，切实增强核心竞争力。

　　充分发挥企业创新主体作用。鼓励和引导企业加大技改、研发投入力度，大力推进技术、产品、品牌、产业组织与商业模式等方面的创新。支持有条件的重点企业建设研发机构，争取新设立20家以上企业技术中心。全面深化产学研用合作，组织实施50项以上区域科技重大项目和100项以上产学研用合作重点项目。

　　不断加强公共创新平台建设。加快发展行业技术创新中心、科技企业孵化器和加速器，完善产权交易、专利代理等创新服务平台。积极参加“6·18”活动，强化与高等院校、科研院所的对接合作。加强创新驿站建设，支持办好中科院海西研究院。推进国家知识产权示范城市建设，力争专利申请量突破1万件。

　　加快构筑闽都人才集聚区。大力建设中国福州海西引智试验区，筹建福州国际人才交流中心、国际人才项目孵化器，建成福州留学人员创业园。实施高层次专业技术人员海内外研修培训计划，举办首届海峡两岸(福州)大学生创业创新大赛。着手组建闽都人才市场，加强人才公寓、国际学校建设。

　　(五)精心打造美丽福州，提高城市宜居宜业和生态文明建设水平。

　　加速构建福州大都市区。切实把马尾新城作为福州新城建设的核心区、先行区和示范区，全面启动马尾快安、马江、长安、琅岐岛，仓山南台岛，长乐营前和连江琯头等片区开发，推进琅岐环岛路、南江滨东段、环南台岛休闲路、长乐滨江滨海大通道等项目建设，动工建设马尾大桥、东部快速通道、乌龙江过江通道等项目，争取琅岐闽江大桥主线通车。加快福清、长乐、闽侯、连江等周边县(市)融入中心城市发展步伐，培育闽清、罗源、永泰等特色功能承载区。编制完成福莆宁同城化总体发展规划，促进福莆宁融合发展、连片繁荣。

　　切实增强中心城市承载能力。动工建设绕城高速公路东南段、福银高速公路闽清(梅溪)互通口等项目，争取建成或基本建成向莆铁路福州段、江阴港铁路支线、可门港铁路支线、福永高速公路、新南港大桥等项目。实施快速路网和重点拥堵节点改扩建工程，完成二环路、斗池路等27条市政道路改造建设。全面完成地铁1号线沿线站点土建工程，动工建设地铁2号线。加快公共停车场建设，鼓励发展立体停车、地下停车。优先发展城市公共交通，新增公交专用道3条，新增更新公交车300辆，新辟和优化公交线路20条，建设智能公交系统，积极创建国家“公交都市”建设示范城市。加快闽江下游防洪排涝等工程建设，健全完善城市防灾减灾体系。推进神华煤港电一体化等重大能源项目建设，争取福清核电站首台机组投产发电。

　　全力打好城乡环境综合整治大会战。编制完善闽江口发展区规划和城市色彩、山体保护等专项规划，争取实现中心城区城市建设用地控规全覆盖，建成城市规划展示馆。认真抓好海峡会展中心、海峡奥体中心周边和东部新城商务办公区、晋安新城等城市组团建设。加快南街改造，实施危旧房(棚屋区)改造230万平方米，综合整治200个老旧住宅小区。精心打造绿城、花城、水城，全面推进城区内河截污和南台岛内河整治，实施内河生态补水工程；提升闽江岸线景观，建设闽江北港驳岸整治一期工程；抓好“点”、“线”、“面”攻坚，加强“四绿”工程建设，造林绿化36.5万亩，改造提升金鸡山公园、金山公园、南公园、江心公园，启动飞凤山、城门山等公园建设，实施二环路、三环路景观线等绿化工程，建设山水路街相衔接的城市“绿道”与慢行系统20公里。争取全面完成市区架空缆线下地。推动“数字城管”系统五城区全覆盖，继续抓好“门前三包”、渣土管理、户外广告清理和集中清理违法建设、治理违法采砂等工作。

　　积极争创国家生态市。大力发展绿色经济、循环经济、低碳经济，实施50项重点节能项目、310项重点减排项目。推进生活垃圾分类收集试点，加快建设餐厨垃圾处理无害化设施、红庙岭垃圾焚烧发电厂二期扩容工程等环保基础设施项目。加强“三江三溪”等重点流域水环境综合整治，实施空气环境质量达标规划。积极倡导“135低碳出行”，推广公共便民自行车服务。强化饮用水源保护区、自然保护区、生态功能区和生态公益林、沿海防护林以及闽江口、乌龙江湿地保护。认真实施重点区域水土流失治理、“碧海银滩”、闽江口生态修复等工程，推进罗源湾污染物排海总量控制试点示范工作。进一步提高土地和海域、岸线等资源节约集约利用水平。探索建立生态资源有偿使用制度，完善生态补偿机制。

　　(六)统筹抓好“三农”工作，扎实推进城镇化和城乡发展一体化。

　　大力发展现代农业。稳定粮食生产，积极发展都市现代农业，培育壮大水产、畜牧、果蔬、食用菌、茶叶、花卉、竹木等特色优势产业。推进福清、长乐、连江等地一批国家级、省级现代农业示范园区、农民创业园建设发展，建设福人花卉市场和园林花卉苗木产业闽侯、永泰基地。扶持联户经营、专业大户、家庭农场，争取亿元产值农业产业化龙头企业突破100家。启动福州茉莉花种植与茶文化系统申报世界重要农业文化遗产工作。加强农技推广、动植物疫病防控、农产品质量监管，实施100项以上农业星火计划项目，创建15家～20家现代农业创新基地，建设2个国家级、7个省级农业标准化示范区。

　　全面推进小城镇和精品示范村建设。进一步推进县(市)城关扩容提质、更新改造。深化小城镇综合配套改革，加快省、市试点小城镇建设发展步伐，打造各具特色的工业强镇、商贸重镇、旅游旺镇和历史文化名镇。继续开展新农村建设“百村竞赛”活动，推进“美丽乡村”建设，加快培育发展一批特色示范村。

　　持续改善农村生产生活条件。加大县(市)区对口协作力度，因地制宜、科学规划、分类指导、因势利导，强化对老少边贫岛地区的政策扶持、资金扶持与项目扶持，增强落后地区“造血功能”。加快推进霍口水库、葫芦门水库等一批重大水利项目建设，新解决60万农村人口饮水安全问题。加强农村环境综合整治，深入实施农村家园清洁行动，新建改造农村公路150公里，新增更新农村客车80辆，新建农村户用沼气池1000口，新建改建农村卫生户厕5000户，争取实现广播电视户户通。广泛开展“春风·春雨·光彩”、“榕商联村”等活动，实施“造福工程”农村危房改造7000户。

　　(七)积极建设文化强市，进一步推动文化大发展大繁荣。

　　大力弘扬闽都文化。加强历史文化名城保护，强化“三山两塔”核心区保护开发，实施朱紫坊、上下杭历史文化街区和烟台山历史风貌区、船政文化遗址群保护修复。规划建设海峡非物质文化遗产生态园、民间传统工艺美术品交易平台，建成东方漆空间创意园。进一步科学开发历史文化名镇(村)资源，发展少数民族民俗文化游等特色项目。

　　不断提升公共文化服务。加快推进海峡图书馆、海峡群艺馆建设，建成市工人文化宫。加强基层公共文化设施建设，构筑城区15分钟、农村30分钟公共文化服务圈。深入开展“四进社区”、“农村电影放映”、“文明小戏进农村”等文化惠民活动。积极培养和引进名家大师，加强文艺精品创作，促进哲学社会科学、新闻出版、广播影视、文学艺术等事业繁荣发展。

　　切实让文明福州持续文明。深入开展社会主义核心价值体系学习教育，大力弘扬民族精神、时代精神。积极普及科学知识。实施公民思想道德素质、志愿服务系统、文明风尚传播、未成年人思想道德建设、农村文明建设、文明城市长效管理等“六个工程”建设，不断提升城乡文明程度与市民文明素质。

　　(八)多办惠民实事，加快建设幸福和谐的“有福之州”。

　　千方百计增加城乡居民就业与收入。深化收入分配制度改革，促进居民收入增长和经济发展同步、劳动报酬增长和劳动生产率提高同步，力争农民收入增速超过城镇居民。积极促进就业，争取城镇新增就业13.3万人，转移农业富余劳动力4.6万人，实施职业技能培训4.8万人次、创业培训5000人次。努力构建和谐劳动关系，依法维护劳动者和企业合法权益。

　　统筹完善城乡社会保障体系。加大社会保险扩面力度，统筹做好城乡居民社会养老保险和被征地农民养老保险工作，探索建立城镇居民大病保险制度，尽快成立四城区社会保险分中心。适时提高城乡低保标准，强化动态管理，确保应保尽保。加强“菜篮子”工程建设，努力维护正常的市场秩序。推进保障房、安置房建设，新开工建设2万套保障性住房，力争建成1万套保障性住房。积极推行“模拟征迁”等和谐征迁新模式，认真解决被征地拆迁群众和退养渔民生产生活出路问题。完善社区居家养老服务长效机制，支持发展社会化养老服务，新建一批农村敬老院，推进老干部活动中心等项目建设。大力发展社会福利、社会救助、社会慈善事业，动工建设市社会福利中心、残疾人康复就业中心。

　　努力办好人民满意的教育。新建和改扩建35所公办幼儿园，探索实施学前教育购买服务模式。完善中小学布局，新建和改扩建30所中小学，扩容中小学学位1万个，全面完成义务教育标准化学校创建任务。创建20所多样化特色化建设试点普通高中。进一步提高市属高校办学水平。推动教育资源配置向农村尤其是边远、贫困、民族乡村倾斜，做好家庭经济困难学生资助和农民工子女入学等工作。大力发展职业教育，建成职业教育公共实训基地。支持发展特殊教育、老年教育、继续教育、社区教育。

　　持续提高人民健康水平。完善省会医疗资源布局，启动市妇幼保健院迁址新建、市二医院内科楼等项目建设。加强基层医疗机构改造提升及设备标准化配置。完善疾病防控、卫生监督、医疗救援、卫生应急和卫生信息体系，创建鼓楼、长乐国家慢性病综合防治示范区和台江宁化等国家级示范社区卫生服务中心。大力扶持中医药事业发展。深入开展爱国卫生运动。认真实施“生育文明·幸福家庭”促进计划，促进人口长期均衡发展。

　　大力推动全民健身与城运同行。加快推进海峡奥体中心“一场三馆”和运动员村建设，抓紧建设、改造一批县(市)区体育场馆。认真备战第十五届省运会和第八届城运会，精心办好世界沙排巡回赛、国际公路自行车赛、十万人健步行等大型赛事和全民健身活动。加强群众健身场所和设施建设，进一步营造全社会关心城运、支持城运、参与城运的浓厚氛围。

　　不断加强和创新社会管理服务。全面推行“135”社区党建工作模式，加强社区网格化服务管理。持续推进“数字福州”建设，完善便民呼叫中心12345系统、“中国福州”门户网站、“福州发布”政务微博群等便民利民公共服务平台。健全社会稳定风险评估、应急处置等机制，完善信访制度和“大调解”工作体系，及时排查调处社会矛盾纠纷。不断巩固提升“平安福州”建设成果，深化综合警务改革，加快建设城市视频监控系统，稳步实施流动人口“居住证”制度，有效防范和依法打击各类违法犯罪活动。加强企业安全生产标准化建设，深入开展安全生产专项整治，坚决防范和遏制重特大安全生产事故。进一步做好“六五”普法和法律援助、法律服务、社区矫正、青少年事务社工等工作。扩大基层民主和群众自治，引导社会组织健康有序发展。做好新形势下的民族宗教工作。坚持军民融合式发展，深化双拥共建，加强国防教育、国防动员、国防后备力量建设及武警现代化建设、海防、人防等工作。三、全面提高新形势下政府自身建设水平

　　各位代表，新的形势、新的任务对政府自身建设提出了新的更高的要求。市人民政府将认真贯彻落实中央和省委、市委改进工作作风、密切联系群众的各项规定，不断提高执行力、公信力和战斗力。

　　切实强化依法行政。进一步增强法治观念，严格按照法定权限和程序行使权力。严格规范行政执法，完善行政复议机制。积极推行公示、咨询、听证等制度。依法接受人大及其常委会的法律监督、工作监督，主动支持政协履行政治协商、民主监督、参政议政职能，积极配合人大、政协开展检查、视察、调研等活动，加强与各民主党派、工商联和各人民团体、无党派人士的沟通联系，提高人大代表建议、批评、意见和政协提案办理的满意率、落实率。

　　全面改进工作作风。深入开展为民务实清廉教育实践活动，完善深入企业、基层常态化服务机制，多做惠民生、谋民利和打基础、利长远的实事好事。大力倡导敢于担当、勇于负责的精神，强化绩效管理、效能建设和岗位考核，全面提高行政效率，让“马上就办”成为广大公务员的行为规范和福州的品牌标志。不断加强学习，提高把握规律、科学决策的能力与水平。切实改进会风文风，精简会议活动和文件简报。进一步简化和规范接待、警卫、出访等工作。

　　时刻保持清正廉洁。扎实推进惩治和预防腐败体系建设，深化反腐倡廉教育和廉政文化建设。加强行政监察，强化领导干部经济责任、财政性资金等审计监督，坚决纠正损害群众利益的不正之风，坚决查处违纪违法行为。严格控制一般性支出，勤俭办一切事业。进一步完善行政权力阳光运行平台，加强政务公开、政府信息公开，深入开展民主评议政风行风等活动，主动接受社会公众和舆论的监督。

　　各位代表，在党的十八大精神指引下，我们将开启福州科学发展跨越发展新的征程。让我们紧密团结在以习近平同志为总书记的党中央周围，在省委、省政府和市委的坚强领导下，进一步敢为、能为、有为，为加快建设开放、文明、和谐、幸福的新福州，率先全面建成小康社会而努力奋斗！
